# Patient perceptions and expectations regarding imaging for metastatic disease in early stage breast cancer

**DOI:** 10.1186/2193-1801-3-176

**Published:** 2014-04-05

**Authors:** Demetrios Simos, Brian Hutton, Ian D Graham, Angel Arnaout, Jean-Michel Caudrelier, Sasha Mazzarello, Mark Clemons

**Affiliations:** Division of Medical Oncology and Department of Medicine, University of Ottawa, Ottawa, Canada; Ottawa Hospital Research Institute, Ottawa, Canada; Department of Epidemiology and Community Medicine, Centre for Practice Changing Research, University of Ottawa, Ottawa, Canada; Department of Surgery, University of Ottawa, Ottawa, Canada; Division of Radiation Oncology and Department of Radiology, University of Ottawa, Ottawa, Canada; Division of Medical Oncology, The Ottawa Hospital Cancer Centre, 501 Smyth Road, Box 900, Ottawa, K1H8L6 Canada

**Keywords:** Imaging, Metastases, Operable breast cancer, Guidelines, Patient perceptions

## Abstract

**Purpose:**

The probability of detecting radiologically evident metastatic disease in asymptomatic women with newly diagnosed operable breast cancer is low. Despite the recommendations of most practice guidelines imaging is still frequently performed. Relatively little is known about what patients believe is important when it comes to radiologic staging.

**Methods:**

Patients with early stage breast cancer who had completed their definitive breast surgery were surveyed about their personal experiences, perceptions, and expectations on the issue of perioperative imaging for distant metastatic disease.

**Results:**

Over a 3 month period, 245 women with primary operable breast cancer completed the questionnaire (87.0% response rate) and 80.8% indicated having had at least one imaging test for distant metastatic disease. These were either of the thorax (72.2%), abdomen (55.9%) or skeleton (65.3%) with a total of 701 imaging tests (average of 3.5 tests per patient imaged) performed. Overall, 57.1% indicated that they would want imaging done if the chance of detecting metastases was ≤10%. Although 80.0% of patients indicated that, “doing whatever their doctor recommended” was important to them, 70.4% also noted that they would be uncomfortable if their physician did not order imaging, even if this was in keeping with practice guidelines.

**Conclusions:**

Most patients with early stage breast cancer recall having imaging tests for distant metastases. Given the choice, most would prefer having imaging performed, even if this is not in line with current guidelines. If patient expectations are, in part, driving excessive imaging, new strategies addressing this are required.

**Electronic supplementary material:**

The online version of this article (doi:10.1186/2193-1801-3-176) contains supplementary material, which is available to authorized users.

## Introduction

Patients with asymptomatic, newly diagnosed early stage breast cancer frequently undergo imaging for distant metastases (Simos et al. 2013; Barrett et al. 2009; Al-Husaini et al. 2008; McWhirter et al. 2007; Puglisi et al. 2005; Gerber et al. 2003; Dillman and Chico 2000; Samant and Ganguly 1999; Ravaioli et al. 1998). Many groups have shown that the probability of detecting such metastases is low (Barrett et al. 2009; Puglisi et al. 2005; Gerber et al. 2003; Dillman and Chico 2000; Samant and Ganguly 1999; Ravaioli et al. 1998; Al-Husaini et al. 2008; Simos et al. 2013) with a recent meta-analysis reporting the median prevalence of metastases as 0.2%, 1.2% and 8% in patients with stage 1, 2 and 3 disease respectively (Brennan and Houssami 2012). As this prevalence is significantly lower than the reported false positive rate of contemporary imaging (Brennan and Houssami 2012; Simos et al. 2013), local (Laing 2012; Myers et al. 2001; Alberta Health Services clinical practice guideline BR-012 version 2 effective July 2012), national (National Comprehensive Network (NCCN) clinical practice guidelines in oncology – Breast Cancer version 1 2014), and international (Senkus et al. 2013) practice guidelines all generally recommend against the routine use of imaging. This issue was recently highlighted (as part of the American Board of Internal Medicine’s “Choosing Wisely Campaign”) by the American Society of Clinical Oncology (ASCO) in their inaugural “Top-5” list for oncology in which the use of staging imaging in asymptomatic patients with early breast cancer was discouraged (Schnipper et al. 2012). This recommendation is in keeping with the spirit of the published guideline recommendations (Myers et al. 2001; Alberta Health Services clinical practice guideline BR-012 version 2 effective July 2012; Laing 2012; National Comprehensive Cancer Network (NCCN) clinical practice guidelines in oncology – Breast Cancer version 1 2014; Senkus et al. 2013).

While guidelines tend to focus on the role of physicians in ordering appropriate investigations (McWhirter et al. 2007), little is known about what patients believe is important when it comes to radiologic staging. We undertook this study to identify patient experiences with as well as their perceptions and expectations on this matter, and to assess whether or not their views are concordant with existing guidelines.

## Methods

### Questionnaire design and target population

A brief questionnaire consisting of eleven questions (Additional file 1) was developed by the study team of medical (DS, MC), radiation (JMC) and surgical (AA) oncologists as well as experts in epidemiology (BH, IG) and knowledge translation (IG). Participants were asked to answer questions about their cancer characteristics, imaging performed around the time of surgery, and their opinion and perceptions around the use of imaging tests. No patient identifiers were used in the data collection process—all the information gathered was based on patient recall.

Our target population included all patients who had completed their definitive breast cancer surgery and were either on adjuvant treatment or on post-treatment follow-up. Patients with metastatic disease and/or who received neoadjuvant chemotherapy and/or who had a previous history of other malignancies were excluded. All participants were patients seen at the Ottawa Hospital Cancer Center (TOHCC) which is a large Canadian academic multidisciplinary cancer centre that routinely sees approximately 1000 new breast cancer patients a year.

### Questionnaire distribution

Paper copies of the questionnaire with an attached letter of introduction explaining rationale and aims of the study were circulated to physicians from the breast cancer disease site group who agreed to enroll patients to this study. Eligible participants were identified their physician during one of their regularly scheduled visits and asked if they would be willing to complete the questionnaire at the conclusion of this visit. This study was approved by the Ottawa Hospital Research Ethics Board.

### Data analysis

All answers were entered into a Microsoft Excel® worksheet for data analysis. The proportion for questions encompassing categorical responses, as well as median values and ranges for continuous measures were calculated. For the number of imaging tests per patient, we used imaging done to look at the most common sites of breast metastases: the skeleton (isotope bone scan, plain x-rays, magnetic resonance imaging (MRI) axial skeleton), thorax (x-ray, computerized tomography (CT)) and liver (ultrasound, CT abdomen/pelvis, magnetic resonance imaging (MRI)). Tables and bar plots were used to summarize the pertinent findings.

## Results

### Patient characteristics

Between March 1 and May 31, 2013, 285 copies of the questionnaire were distributed. A total of 248 completed questionnaires were returned (87.0%). Three questionnaires were excluded from the final analysis (N = 245) as the respondents indicated that they had not yet had their definitive breast cancer surgery (all 3 indicated they were on neoadjuvant chemotherapy at the time). Self-reported patient and disease characteristics of the 245 respondents that were included in the final analysis are shown in Table 1. Median patient age was 59 years (range 27-88) and time from breast surgery to completing the questionnaire was < 6 months in 93 (38.0%), 6-12 months in 50 (20.4%), and > 12 months in 94 (38.4%). Disease stage was reported by 197/245 (80.4%) respondents: 88 (44.7%) were stage 1, 69 (35.0%) stage 2, and 40 (20.3%) stage 3.

**Table 1:** **Patient reported baseline variables**

Respondents		245
Median age (range)		59 (27-88)
Method of cancer detection	Symptomatic	161 (65.7%)
	Screen	81 (33.1%)
	Not answered	3 (1.2%)
Time from surgery to completion of questionnaire	<6 months	93 (38.0%)
	6-12 months	50 (20.4%)
	>12 months	94 (38.4%)
	Not answered	8 (3.3%)
Stage of breast cancer	1	88 (35.9%)
	2	69 (28.2%)
	3	40 (16.3%)
	Unknown	48 (19.6%)
Hormone receptor positive breast cancer	Yes	158 (64.5%)
	No	27 (11.0%)
	Don’t know	52 (21.2%)
	Not answered	8 (3.3%)
Human epidermal growth factor receptor (HER-2) positive breast cancer	Yes	54 (22.0%)
	No	89 (36.3%)
	Don’t know	87 (35.5%)
	Not answered	15 (6.1%)
Triple negative breast cancer	Yes	15 (6.1%)
	No	114 (46.5%)
	Don’t know	85 (34.7%)
	Not answered	31 (12.7%)
Lymph node positive breast cancer	Yes	103 (42.0%)
	No	124 (50.6%)
	Don’t know	9 (3.7%)
	Not answered	9 (3.7%)

Legend: <: less than; >: greater than.

Overall, 198 (80.8%) respondents reported having at least one imaging test for metastatic disease (Table 2). The total number of imaging tests recalled by all respondents was 701 for a median of 3 (range 0-11) and an average of 3.5 imaging tests per patient imaged. Of the 701 total imaging tests performed, 300 (42.8%) were of the thorax, 213 (30.4%) were of the abdomen, and 188 (26.8%) were of the skeleton. Overall, 476 of all imaging tests (67.9%) were performed pre-operatively and 225 (32.1%) post-operatively.

**Table 2:** **Details of patient reported imaging**

**A: Summary of patient reported perioperative imaging for distant metastases**				
Patients reporting at least one imaging test for metastatic disease (%)	198 (80.8%)			
Reported imaging by site (% of total patients):				
Thorax	177 (72.2%)			
Abdomen	137 (55.9%)			
Skeleton	160 (65.3%)			
Total imaging tests by site (% of total imaging):				
Thorax	300 (42.8%)			
Abdomen	213 (30.4%)			
Skeleton	188 (26.8%)			
Total imaging tests (# per patient imaged)	701 (3.5)			
**B: Imaging details**				
	*Stage 1 (n = 88)*	*Stage 2 (n = 69)*	*Stage 3 (n = 40)*	*Unknown Stage (n = 48)*
**Thoracic imaging:**				
Reported in (%)	52 (59.1%)	55 (79.7%)	32 (80.0%)	38 (79.2%)
Total # of imaging tests of chest (% of total of 300)	82 (27.3%)	91 (30.3%)	59 (19.7%)	68 (22.7%)
Imaging details:				
CXR only (%)	25 (28.4%)	29 (42.0%)	11 (27.5%)	15 (31.3%)
CT-chest only (%)	9 (10.2%)	4 (5.8%)	3 (7.5%)	3 (6.3%)
CXR + CT-chest (%)	18 (20.5%)	22 (31.9%)	18 (45.0%)	20 (41.7%)
**Abdominal imaging:**				
Reported in (%)	38 (43.2%)	44 (63.8%)	29 (72.5%)	26 (54.2%)
Total imaging tests of abdomen (% of total of 213)	61 (28.6%)	60 (28.2%)	51 (23.9%)	41 (19.2%)
Imaging details:				
US only (%)	23 (26.1%)	28 (40.6%)	11 (27.5%)	12 (25.0%)
CT only (%)	1 (1.1%)	7 (10.1%)	4 (10%)	4 (8.3%)
MRI only (%)	1 (1.1%)	1 (1.4%)	1 (2.5%)	0 (0%)
Two or more of US/CT/MRI (%)	13 (14.8%)	8 (11.6%)	13 (32.5%)	10 (20.8%)
**Skeletal imaging:** (all reported isotope bone scan)				
Reported in (%)	45 (51.1%)	49 (71.0%)	35 (87.5%)	31 (64.6%)

Legend: CXR: chest x-ray; CT: computerized tomography; MRI: magnetic resonance imaging; US: ultrasonography; %: percentage.

Imaging of the thorax was reported by 177 patients (72.2% overall; 52 stage 1 (59.1%), 55 stage 2 (79.7%), 32 stage 3 (80.0%), and 38 of unknown stage (79.2%)). The most common imaging modalities for the thorax were chest x-ray (80 patients), CT scan (19 patients) or both (78 patients). Imaging of the abdomen was reported by 137 patients (55.9% overall; 38 stage 1 (43.2%), 44 stage 2 (63.8%), 29 stage 3 (72.5%), 26 of unknown stage (54.2%)). The most common imaging modalities for the abdomen were ultrasound (74 patients), CT scan (16 patients), MRI (3 patients), or a combination of these (44 patients). Imaging of the skeleton was reported by 160 patients (65.3% overall; 45 stage 1 (51.1%), 49 stage 2 (71.0%), 35 stage 3 (87.5%), 31 of unknown stage (64.6%)) patients. All respondents reported having an isotope bone scan.

### Acceptable thresholds to perform imaging as reported by patients

**Figure 1:**
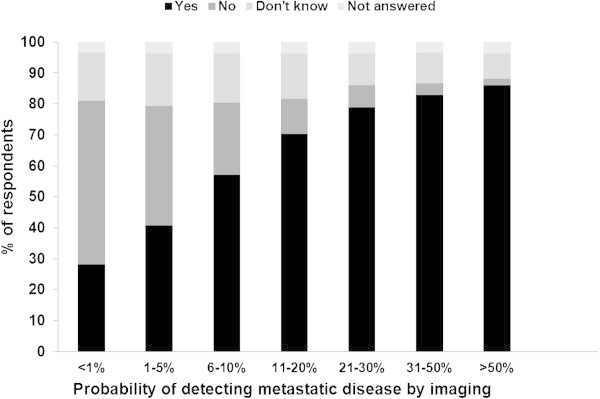
**Patient reported imaging thresholds (N = 245).**

Participants were given a range of probabilities of detecting metastatic disease using imaging and asked to provide a response (“yes”, “no”, or “don’t know”) for the range they felt imaging would be acceptable and expected. The probabilities listed were as follows: <1%, 1-5%, 6-10%, 11-20%, 21-30%, 31-50% and >50%. While 69/245 (28.2%) of patients indicated that they would want imaging even if the chance of metastatic disease was <1%, the majority (140/245; 57.1%) indicated that a probability of detecting distant metastatic disease of between 6-10% (Figure 1).

### Patient reported factors of importance relating to staging imaging

**Figure 2:**
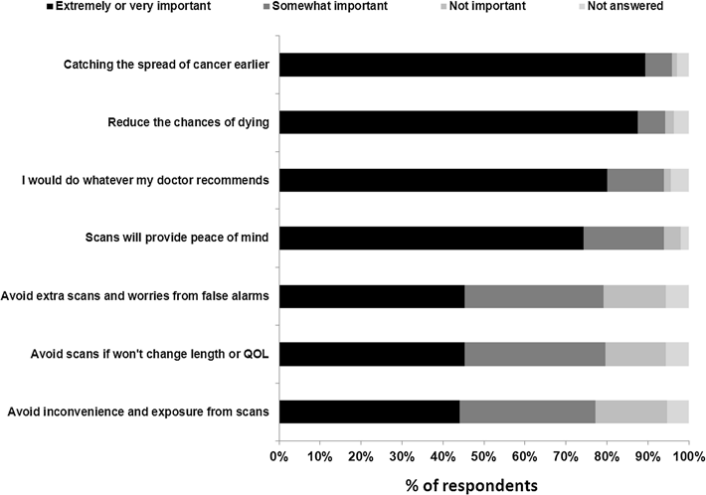
**Patient reported factors of importance (N = 245), QOL: quality of life.**

Patients were asked to indicate how important seven different statements related to staging imaging are to them using a predefined scale consisting of the following choices: extremely important, very important, somewhat important, or not important (Figure 2). Of these 7 statements, 4 were felt by the majority of patients to be either extremely important or very important to them. These were: “catching the spread of cancer to other parts of the body early” (219/245; 89.4%), “reducing the chances of dying from breast cancer” (211/245; 86.1%), “I would do whatever my doctor recommends” (196/245; 80.0%), and “scans will provide peace of mind” (182/245; 74.3%). The remaining 3 statements were deemed somewhat important or not important by the majority of respondents.

### Patient reported level of comfort if imaging based on local guidelines

**Figure 3:**
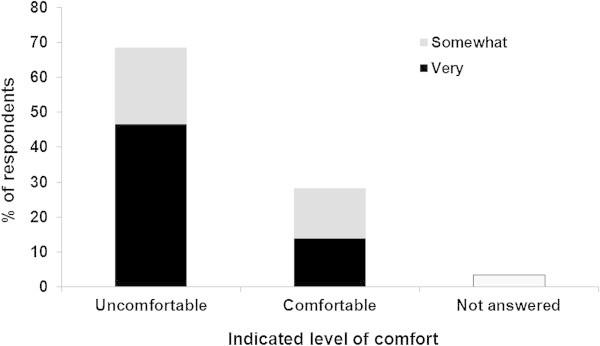
**Patient reported level of comfort if imaging not ordered (N = 245).**

Participants were asked to indicate how comfortable they would be if their physician, in concordance with the local Cancer Care Ontario guideline (Myers et al. 2001), did not perform imaging for metastatic disease (Figure 3). Overall, 168/245 (68.6%) of respondents indicated they would feel, “uncomfortable” with this recommendation. Of the 196 (80.0%) who indicated that doing whatever their physician recommends is either extremely or very important to them, 138/196 (70.4%) indicated that they would be uncomfortable if their physician did not order imaging to look for metastatic disease.

## Discussion

The challenges around controlling excessive staging imaging for metastatic disease in asymptomatic early stage breast cancer are well recognised (McWhirter et al. 2007; Puglisi et al. 2005; Gerber et al. 2003; Dillman and Chico 2000; Simos et al. 2013; Schnipper et al. 2012) and despite guidelines recommending against routine imaging is still frequently over utilised (Barrett et al. 2009; McWhirter et al. 2007; Puglisi et al. 2005; Gerber et al. 2003; Dillman and Chico 2000; Samant and Ganguly 1999; Ravaioli et al. 1998; Al-Husaini et al. 2008; Simos et al. 2013). While most reports focus on diagnostic accuracy of the imaging test (the balance between true and false positive detection rates), (Barrett et al. 2009; Puglisi et al. 2005; Simos et al. 2013; Brennan and Houssami 2012), relatively few have attempted to evaluate the performance of an imaging test on patient outcomes including the potential for harm both to the patient (e.g. anxiety, delays in treatment, out of pocket costs) and to the health care system (e.g. costs of imaging, delayed access to care) (Gerber et al. 2003; Schnipper et al. 2012; Morris et al. 2009). The rates of breast cancer imaging, especially the use of more advanced imaging modalities are on the rise as are their associated costs (Mariotto et al. 2011; Dinan et al. 2010; Crivello et al. 2013; Gold et al. 2013). This specific issue was recently highlighted by the American Society of Clinical Oncology (ASCO) in their recent “Top-5” list for “Choosing Wisely” in oncology (Schnipper et al. 2012). While strategies to promote adherence with guidelines include widespread guideline dissemination (Davis and Taylor-Vaisey 1997), physician interventions (McWhirter et al. 2007), there is little evidence that these strategies result in sustained practice changes (Graham et al. 2013; Grimshaw et al. 2012). Interestingly, relatively little is known about what patients believe is important when it comes to radiologic staging and to our knowledge this is the first study to report what patient expectations and beliefs are with respect to staging imaging for distant metastatic disease.

In this study, patients recalled having a significant amount of imaging performed. Overall, 80.2% of all respondents indicated having at least one imaging test for an average of 3.5 imaging tests per patient imaged with the majority of all imaging done in the pre-operative setting (67.9%). Although these data are based completely on recall, and not verified with the patient record, they are in keeping with the findings of a recent retrospective review at the same centre (Simos et al. 2013). In that study 167 of the 200 patients reviewed (83.5%) had at least one imaging test in the perioperative period and the number of imaging tests per patient imaged was 3.8 with 61.0% being performed pre-operatively (Simos et al. 2013). The similarity of findings between these two studies suggest that although the imaging reported in this study is all based on recall, it is also a reasonably accurate representation of what was actually done and consistent with the over-imaging that is reported in the literature (McWhirter et al. 2007; Puglisi et al. 2005; Gerber et al. 2003; Dillman and Chico 2000; Samant and Ganguly 1999; Ravaioli et al. 1998; Al-Husaini et al. 2008). These findings are in excess of the recommendations of our provincial guideline (Cancer Care Ontario) which recommends stage 2 patients should only have a bone scan and that only stage 3 patients should have imaging of the thorax, abdomen and skeleton (Myers et al. 2001).

The timing of imaging reported in this study (and also our previous review (Simos et al. 2013)) suggests most of the staging imaging to look for distant metastases is done in the pre-operative period. This is an interesting finding given that our provincial guideline recommends staging imaging based on post-operative pathologic stage (Myers et al. 2001). The reasons why almost twice as many patients are having radiologic staging done before rather than after their surgery are not clear from this study, but personal communication with our local surgical colleagues revealed reasons as; 1) a significant proportion of patients already had their staging tests ordered by their primary care physician 2) the surgeon’s desire to potentially reduce the wait time for medical and radiation oncology consultations after surgery 3) the potential to identify metastatic patients early to avoid surgery as primary therapy, and 4) requests from patients. Clearly, strategies to promote knowledge translation and adherence to practice guidelines need to target health care providers at all levels, including the primary care physician, surgeon, and oncologists.

Participants were asked about what the threshold for having distant metastases should be to justify the use of imaging. The majority of patients believed that imaging should be performed if the chance of detecting distant metastases was between 6-10%. In other words, most patients believe that a likelihood of detecting metastatic disease of >5% and this is an interesting finding in the context of reported likelihoods of detecting metastatic disease. Recently, a large meta-analysis reported that the median prevalence of detecting radiologically evident metastases in asymptomatic patients with stage 1, 2 and 3 disease were 0.2%, 1.2%, and 8.0%, respectively (Brennan and Houssami 2012). As the likelihoods of detecting occult metastases using imaging in stage 1 and 2 disease by are less than the 5% threshold reported by the patients completing this study, it can be reasoned that most patients who took part in our study do not believe imaging is indicated for stage 1 or 2 disease. Interestingly, our provincial guideline (Cancer Care Ontario guideline) sets a 1% prevalence cut-off value as the threshold for consideration of imaging (Myers et al. 2001).

The majority of respondents indicated that “catching the spread of cancer to other parts of the body early”, “reducing the chances of dying from breast cancer”, and “providing peace of mind” were very important or extremely important to them. However, this is quite different from the primary reason why most physicians order imaging, which is for detecting overt metastases in the asymptomatic setting. In reality, detection of metastatic disease neither “catches the spread of cancer to other parts of the body early” nor “reduces the chances of dying from breast cancer”. However, if imaging is being performed to “rule out in the presence of metastatic disease” it is clearly evident that many patients will relapse despite the presence of “normal” imaging. Further highlighting this discrepancy between physician and patient perceptions and expectations is that the overwhelming majority of patients indicated that they would feel uncomfortable if their physician did not order imaging to look for metastatic disease in their circumstance, even if this physician choice was in keeping with the evidence based guidelines. Only 13.9% indicated that they would feel very comfortable with this recommendation, and this is despite 80.0% of respondents indicating that they would do whatever their doctor recommends.

There are limitations to this study. Given that this was a questionnaire based study at a single cancer centre, there is always the issue of recall bias, social response bias, leading to incomplete or erroneous data. As we deliberately did not capture patient identification data, we were unable to verify the concordance between patient recall and their medical record. However, the rate and timing of imaging reported by patients in this study are very similar those reported in our prior retrospective review (Simos et al. 2013). Furthermore, we have not included confounding variables such as the use of breast imaging as this is used for locoregional detection of cancer spread and not for the detection of distant metastases.

In conclusion, we have demonstrated that patients with early stage primary operable breast cancer recall having undergone a significant amount of imaging. More importantly however, we have demonstrated that patients’ perceptions and expectations are generally not in keeping with guideline recommendations not to image. The reasons behind this disconnect are unclear but clearly we need to improve the way patients are informed about the potential benefits and harms of imaging. Further work is needed on how to successfully change such practice in light of the recently published American Society of Clinical Oncology (ASCO) “Top-5” list in Oncology which identified excessive imaging in early stage breast cancer as an unnecessary and potentially harmful practice.

### Consent

All patients provided consent prior to completing the study questionnaire.

## Electronic supplementary material

Additional file 1: **Study questionnaire.** (DOC 50 KB)

## References

[CR1] Alberta Health Services clinical practice guideline BR-012 version 2 effective July 2012 2014. http://www.albertahealthservices.ca/hp/if-hp-cancer-guidebr012-staging-investigations.pdf. Accessed 27 February 2014

[CR2] Al-Husaini H, Amir E, Fitzgerald B, Wright F, Dent R, Fralick J, Clemons M (2008). Prevalence of overt metastases in locally advanced breast cancer. Clin Oncol (R Coll Radiol).

[CR3] Barrett T, Bowden DJ, Greenberg DC, Brown CH, Wishart GC, Britton PD (2009). Radiological staging in breast cancer: which asymptomatic patients to image and how. Br J Cancer.

[CR4] Brennan ME, Houssami N (2012). Evaluation of the evidence on staging imaging for detection of asymptomatic distant metastases in newly diagnosed breast cancer. Breast.

[CR5] Crivello ML, Ruth K, Sigurdson ER, Egleston BL, Evers K, Wong YN, Boraas M, Bleicher RJ (2013). Advanced imaging modalities in early stage breast cancer: preoperative use in the Unites States Medicare population. Ann Surg Oncol.

[CR6] Davis D, Taylor-Vaisey A (1997). Translating guidelines into practice: a systematic review of theoretic concepts, practical experience and research evidence in the adoption of clinical practice guidelines. CMAJ.

[CR7] Dillman RO, Chico S (2000). Radiologic tests after a new diagnosis of breast cancer. Eff Clin Pract.

[CR8] Dinan MA, Curtis LH, Hammill BG, Patz EF, Abernethy AP, Shea AM, Schulman KA (2010). Changes in the use and costs of diagnostic imaging among Medicare beneficiaries with cancer, 1996-2006. JAMA.

[CR9] Gerber B, Seitz E, Muller H, Krause A, Reimer T, Kundt G, Friese K (2003). Perioperative screening for metastatic disease is not indicated in patients with primary breast cancer and no clinical signs of tumor spread. Breast Cancer Res Treat.

[CR10] Gold LS, Buist DSM, Loggers ET, Etzioni R, Kessler L, Ramsey SD, Sullivan SD (2013). Advanced diagnostic breast cancer imaging: variation and patterns of care in Washington state. J Oncol Pract.

[CR11] Graham ID, Tetroe J, Gagnon M (2013). Knowledge Dissemination.

[CR12] Grimshaw JM, Eccles MP, Lavis JN, Hill SJ, Squires JE (2012). Knowledge translation of research findings. Implement Sci.

[CR13] Laing K (2012). Eastern Health Care Cancer Care clinical practice guidelines staging of primary breast cancer guideline summary.

[CR14] Mariotto AB, Yabroff KR, Shao Y, Feuer EJ, Brown ML (2011). Projections of the cost of cancer care in the United States: 2010-2012. J Nat Cancer Inst.

[CR15] McWhirter E, Yogendran G, Wright F, Dranitsaris G, Clemons M (2007). Baseline radiological staging in primary breast cancer: impact of educational interventions on adherence to published guidelines. J Eval Clin Pract.

[CR16] Morris PG, O’Connor MO, Rafferty CO, Sheikh R, Gray J, McDermott R, Boyle T, Kennedy MJ (2009). The excessive cost of baseline diagnostic imaging in early breast cancer. Ir Med J.

[CR17] Myers RE, Johnston M, Pritchard K, Levine M, Oliver T, and the members of the Breast Cancer Disease Site Group of the Cancer Care Ontario Practice Guidelines Initiative (2001). Baseline staging tests in primary breast cancer: a practice guideline. CMAJ.

[CR18] National Comprehensive Cancer Network (NCCN) clinical practice guidelines in oncology – Breast Cancer version 1 2014. http://www.nccn.org/professionals/physician_gls/pdf/breast.pdf. Accessed 27 February 2014

[CR19] Puglisi F, Follador A, Minisini AM, Cardellino GG, Russo S, Andreetta C, Di Terlizzi S, Piga A (2005). Baseline staging tests after a new diagnosis of breast cancer: further evidence of their limited indications. Ann Oncol.

[CR20] Ravaioli A, Tassinari D, Pasini G, Polselli A, Papi M, Fattori PP, Pasquini E, Masi A, Alessandrini F, Canuti D, Panzini I, Drudi G (1998). Staging of breast cancer: what standards should be used in research and clinical practice. Ann Oncol.

[CR21] Samant R, Ganguly P (1999). Staging investigations in patients with breast cancer. Arch Surg.

[CR22] Schnipper LE, Smith TJ, Raghavan SD, Blayney DW, Ganz PA, Mulvey TM, Wollins DS (2012). American Society of Clinical Oncology identifies 5 key opportunities to improve care and reduce costs: the top five list for oncology. J Clin Oncol.

[CR23] Senkus E, Kyriakides S, Penault-Llorca F, Poortmans P, Thompson A, Zackrisson S, Cardoso F, on behalf of the members of the ESMO Guidelines Working Group (2013). Primary breast cancer: ESMO clinical practice guidelines for diagnosis, treatment and follow-up. Ann Oncol.

[CR24] Simos D, Hutton B, Fergusson D, Clemons M (2013). Has the ASCO top-5 changed radiologic staging practices amongst physicians who treat breast cancer?. J Clin Oncol.

