# Supplementary material for: Patient perceptions and expectations regarding imaging for metastatic disease in early stage breast cancer
Source: Springerplus. 2014 Apr 5;3:176. doi: 10.1186/2193-1801-3-176 (PMC4000356; doi:10.1186/2193-1801-3-176)
Supplement: Supplementary file 1 — Additional file 1: Study questionnaire. (DOC 50 KB) [file 40064_2014_887_MOESM1_ESM.doc]

**Additional file 1**

Study questionnaire:

**Please indicate or fill in the answer that best applies to you.**

**1. How old were you when you were diagnosed with breast cancer: ___________**

**2. How was your breast cancer first found?**

a) Originally found by yourself (i.e. you noticed a lump, change in skin or nipple, pain etc)

b) On screening mammogram ordered by a heath care professional

c) Examination by your health care professional when you were not aware of any abnormality

**3. After your biopsy that diagnosed your breast cancer, but before your surgery to remove the cancer from your breast(s), did you have any of the following imaging tests (scans) done? Please indicate all you remember having.**

a) Mammogram

i) Yes ii) No iii) Don’t know

b) Ultrasound of breasts

i) Yes ii) No iii) Don’t know

c) MRI of breasts

i) Yes ii) No iii) Don’t know

d) Chest x-ray

i) Yes ii) No iii) Don’t know

e) Ultrasound of abdomen

i) Yes ii) No iii) Don’t know

f) Bone scan

i) Yes ii) No iii) Don’t know

g) CT scan of your chest

i) Yes ii) No iii) Don’t know

h) CT scan of abdomen

i) Yes ii) No iii) Don’t know

i) CT scan of pelvis

i) Yes ii) No iii) Don’t know

j) Other: please specify_________________________________

**4. How long ago was your surgery?**

a) Less than 6 months ago

b) Between 6 and 12 months ago

c) More than 12 months ago

**5. After the surgery to remove the cancer from your breast(s) but before starting any further treatment (such as radiation therapy, chemotherapy, hormonal therapy) did you have any of the following imaging tests (scans) done to see if your cancer had spread? Please indicate all you remember having.**

a) Chest x-ray

i) Yes ii) No iii) Don’t know

b) Ultrasound of abdomen

i) Yes ii) No iii) Don’t know

c) Bone scan

i) Yes ii) No iii) Don’t know

d) CT scan of chest

i) Yes ii) No iii) Don’t know

e) CT scan of abdomen

i) Yes ii) No iii) Don’t know

f) CT scan of pelvis

i) Yes ii) No iii) Don’t know

g) CT Scan of head

i) Yes ii) No iii) Don’t know

h) MRI of abdomen

i) Yes ii) No iii) Don’t know

i) Other: please specify_______________________________

**6. At the time of your diagnosis, did you have?**

a) Stage 1 breast cancer

b) Stage 2 breast cancer

c) Stage 3 breast cancer

d) Don’t know

**7. For each of the following, please indicate whether it applies to your breast cancer.**

a) Hormone receptor positive (i.e. you breast cancer had estrogen and/or progesterone receptors on the cancer cells)

i) Yes ii) No iii) Don’t know

b) Her-2 positive (i.e. your breast cancer had high levels of Her-2 on the cancer cells)

i) Yes ii) No iii) Don’t know

c) Triple negative (i.e. your breast cancer did not have estrogen and progesterone receptors or high expression of Her-2 on the cancer cells)

i) Yes ii) No iii) Don’t know

d) Lymph node positive (i.e. your breast cancer was found in the lymph nodes taken from your arm pit during your surgery)

i) Yes ii) No iii) Don’t know

**8. Question 8 has 2 parts: part a and b.**

**Let’s assume that there are 100 women with your stage of breast cancer and that all of these women had the scans listed in the table below just before or after their surgery to remove their breast cancer. These scans were ordered by their physician(s) in order to look for breast cancer that may have already spread elsewhere in their body.**

***[CLARIFICATION for Question 8:***

*In question #8 [both part a) and b)], we are asking you to give us your best guess/estimate. We do not expect you to know the “right” answer. By asking you about this question we are trying to get a feel about how good you (the patient) think these scans are at detecting cancer elsewhere in the body of a woman with your exact stage/type of breast cancer.*

*Part a) asks you to look at each test individually. For example if you think the bone scan is a perfect test, then it would detect the cancer elsewhere in all 100 women just like you (100%) and your answer would be 100/100. The same applies to the other tests listed in the table. If you don’t think the test is perfect, then the number will naturally be less than 100 as it will miss picking up the cancer in the area of the body it scans.*

*Part b), assumes you have done all the tests listed in part a) and asks you to guess/estimate what you think the chance of finding cancer in at least one other part of the body would be if all three tests were done at the same time.]*

a) For each of the following scans, please indicate in how many women (out of 100) you would expect each scan to detect cancer elsewhere?

| **Imaging Test (Scan)** | **Detection of Disease Elsewhere (out of 100)** |
| --- | --- |
|  |  |
| Bone scan | /100 |
| Abdominal ultrasound | /100 |
| Chest x-ray | /100 |

b) The scans listed in the table above each look at a specific part of the body that breast cancer is most likely to be found in if it has spread. For example, the bone scan looks for cancer that has spread to bones but cannot tell your doctor whether the cancer has spread to the liver or lungs. Once the cancer has spread beyond the breast and lymph nodes of the arm pit, it can no longer be cured and the treatment needs to be modified accordingly. This is considered stage 4 disease.

Knowing this, out of 100 women with your stage of breast cancer who go on to have the scans listed in the table above, in how many would cancer be found elsewhere in their body?

_______________________ out of 100

**9. In the table below are listed some hypothetical probabilities (i.e. the chances) of finding breast cancer elsewhere in your body with imaging tests (scans). For each probability please indicate whether you would want your physician to recommend scans to look for breast cancer elsewhere in the body:**

| **Probability (Chance) of Finding Cancer Elsewhere in My Body** | **Should my Physician Recommend Scans** | | |
| --- | --- | --- | --- |
|  | **Yes** | **No** | **Don’t Know** |
| Less than 1 out of 100  (Less than 1%) |  |  |  |
| Between 1 and 5 out of 100  (1-5%) |  |  |  |
| Between 6 and 10 out of 100  (6-10%) |  |  |  |
| Between 11 and 20 out of 100  (11-20%) |  |  |  |
| Between 21 and 30 out of 100  (21-30%) |  |  |  |
| Between 31 and 50 out of 100  (31-50%) |  |  |  |
| More than 50 out of 100  (More than 50%) |  |  |  |

**10. All medical tests, imaging tests (scans) included, are not perfect. There are always benefits and risks associated with their use. Deciding on whether or not to agree to a scan which looks for breast cancer elsewhere in your body (just before or just after your surgery to remove the breast cancer) depends on a number of factors such as; the likelihood the cancer has actually spread, the ability of the scan to actually pick-up the cancer that has spread, the risks associated with the scan itself (i.e. radiation exposure, patient anxiety while waiting for the results) and physician preference to name a few.**

**For each of the following statements please indicate how important each one is to you:**

| **Statement** | **Importance** | | | |
| --- | --- | --- | --- | --- |
|  | **None** | **Somewhat** | **Very** | **Extremely** |
| Scans will provide peace of mind |  |  |  |  |
| Catching the spread of cancer to other parts of the body early |  |  |  |  |
| Reducing the chances of dying from breast cancer |  |  |  |  |
| Avoiding extra scans and worry from false alarms |  |  |  |  |
| Avoiding scans if the results will not change the length or quality of life |  |  |  |  |
| Avoid the inconvenience and exposure to radiation from scans when the results will not change how my cancer is managed |  |  |  |  |
| I would do whatever my doctor recommends |  |  |  |  |

**11. If the Cancer Care Ontario clinical practice guidelines did not support use of imaging tests (scans) in your particular situation, how comfortable would you be if your doctor followed these practice guidelines and did not send you for scans to look for cancer elsewhere in your body?**

a) Very comfortable

b) Somewhat comfortable

c) Somewhat uncomfortable

d) Very uncomfortable

If you wish to explain your answer, please do so below:

____________________________________________________________________________________________________________________________________________________________
